# Astaxanthin ameliorates ferric nitrilotriacetate-induced renal oxidative injury in rats

**DOI:** 10.3164/jcbn.16-114

**Published:** 2017-06-15

**Authors:** Yasumasa Okazaki, Shigeru Okada, Shinya Toyokuni

**Affiliations:** 1 Department of Pathological Research, Okayama University Graduate School of Medicine, Dentistry and Pharmaceutical Sciences, Okayama, Okayama 700-8558, Japan; 2 Department of Pathology and Biological Responses, Nagoya University Graduate School of Medicine, 65 Tsurumai, Showa-ku, Nagoya 466-8550, Japan; 3 Department of Anti-Aging Food Sciences, Okayama University Graduate School of Medicine, Dentistry, and Pharmaceutical Sciences, 2-5-1 Shikata-cho, Kita-ku, Okayama 700-8558, Japan

**Keywords:** astaxanthin, ferric nitrilotriacetate, oxidative stress, vitamin E

## Abstract

Daily intake of vegetables can reduce the risk of cancer and lifestyle-related diseases. However, supplementary intake of β-carotene alone has been reported to increase the risk of lung cancer in male cigarette smokers and people who were exposed to asbestos. The mechanism of the antioxidative properties of carotenoids *in vivo*, especially under oxidative stress conditions, still remains unclear. To investigate the antioxidant properties of dietary compounds, we examined the effects of chemically modified astaxanthin (Ax-C-8) using a rat model of ferric nitrilotriacetate (Fe-NTA)-induced renal oxidative injury. Ax-C-8 demonstrated lethally toxic effects on the rats in a dose-dependent manner. Following supplementation with Ax-C-8 (0.02%, w/w) for 30 days, the rats were euthanized 1, 4 and 24 h after injection of Fe-NTA. After 4 h, Ax-C-8 pretreatment suppressed the elevation of creatinine and blood urea nitrogen and protected the rats from renal tubular necrosis and the formation of 4-hydroxy-2-nonenal-modified proteins. After 24 h, pretreatment with Ax-C-8 maintained the renal antioxidant enzyme levels and renal tubules. Here, we demonstrate the antioxidant effects of Ax-C-8 against Fe-NTA-induced oxidative injury in rats receiving a regular diet. These data suggest that dietary intake of astaxanthin may be useful for the prevention of renal tubular oxidative damage.

## Introduction

Lifestyle-related pathological conditions such as obesity, diabetes mellitus, hypertension and cardiovascular disease are causatively associated with oxidative stress. It is a well-known fact that the intake of fruits and vegetables reduces the risk of developing cancer. Thus, dietary intake of antioxidative foods in daily life may decrease the burden of oxidative stress and be beneficial for the promotion of health.^(1,2)^ Among the bioavailable compounds in foods, carotenoids are common compounds that are responsible for most of the yellow to red color of fruits and flowers. The physiological roles of carotenoids in animals involve cellular growth, differentiation and the prevention of oxidative stress. The β-Carotene and Retinol Efficacy Trial (CARET) failed to demonstrate preventive effects, and an increase in lung cancer onset and mortality was observed in the high-risk population, which consisted of smokers, former smokers and workers who were exposed to asbestos.^(3,4)^ Although dietary compounds with antioxidative properties are well known to exhibit bioactivity, the *in vivo* bioavailability of each element still remains unclear.

Recently, many types of functional foods that are thought to play a supportive role in promoting health have entered the market. However, only a small fraction of these have been evaluated using established methods, and evidence-based review guidelines for qualified functional foods are emerging to protect citizens from harmful information.^(5)^ It will be necessary in the future to provide evidence that antioxidative foods are really functional using a scientific approach. In this study, the biological effects of astaxanthin (Ax) were examined using an animal model. Ax is enzymatically synthesized from the oxidation of β-carotene in plants and microorganisms,^(6)^ and this natural pigment is found in vegetables, salmon and shrimp. Ax has been shown to possess efficient antioxidant capacity, which is approximately 2-fold higher than β-carotene, by entering the phospholipid membrane and predicted to form hydrogen bounding with tocotrienol *in vitro*.^(7–9)^ Previous studies have demonstrated that Ax protects against tissue injury from exercise, aging and diabetes mellitus, which are related to oxidative stress in rodents.^(10–13)^

To clarify the potential mechanism of Ax in oxidative stress, we employed a chemically modified Ax C-8 ester (Ax-C-8) and an animal model of oxidative renal tubular damage induced by ferric nitrilotriacetate (Fe-NTA).^(14)^ Intraperitoneal (i.p.) administration of this iron chelate to rats and mice catalyzes the generation of ROS and accelerates lipid peroxidation in the kidney,^(15)^ finally leading to a high incidence of renal cell carcinoma.^(16)^ Increases in malondialdehyde, 2-thiobarbituric acid-reactive substances (TBARS),^(15)^ 4-hydroxy-2-nonenal (HNE)-modified proteins,^(17)^ and 8-hydroxy-2'-deoxyguanosine (8-OHdG) were observed in this model.^(18)^ Vitamin E,^(19,20)^ lycopene,^(21)^ lactoferrin,^(22)^ curcumin,^(23)^ and a wide variety of compounds have been reported to exhibit protective effects in this carcinogenesis model.

In the present study, we reveal that Ax-C-8 has advantageous effects on oxidative stress-induced renal injury *in vivo*. The potential mechanisms and their implications will be discussed.

## Materials and Methods

### Chemicals

Iron nitrate enneahydrate, nitrilotriacetic acid, oxidized and reduced glutathione, glutathione reductase, and hydrogen peroxide were purchased from Wako (Osaka, Japan). Nicotinamide adenine dinucleotide phosphate reduced tetrasodium salt, glucose-6-phosphate, 1-chloro-2,4-dinitrobenzene (CDNB), 5,5'-dithio-*bis*-2-nitrobenzoic acid (DTNB), bovine serum albumin, trichloroacetic acid, and Tween 20 were purchased from Sigma (St. Louis, MO). 2-Thiobarbituric acid (TBA) was purchased from Merck (Darmstadt, Germany). The basal MF diet (powder) and Vitamin E (VE)-deficient diet were purchased from Oriental Yeast (Tokyo, Japan). The VE-stripped corn oil was purchased from TAMA Biochemicals (Tokyo, Japan). The BCA assay kit was purchased from Pierce (Rockford, IL). The Ax-C-8 was a kind gift from the Institute for Health Care Science, Suntory (Osaka, Japan). Normal goat serum was purchased from Vector Laboratories (Burlingame, CA), and Histofine Simple Stain rat Max-PO (multi) was purchased from Nichirei Biosciences (Tokyo, Japan). Liquid DAB was purchased from DAKO Japan (Tokyo, Japan). The two monoclonal antibodies against 8-OHdG (N45.1) and HNE-modified proteins (HNE-J2) were from the Japan Institute for the Control of Aging (Shizuoka, Japan). All of the other chemicals were of the highest quality available from Wako (Osaka, Japan).

### Preparation of Fe-NTA solution

Briefly, nitrilotriacetic acid (NTA) and iron nitrate enneahydrate were dissolved in distilled water. The mixture of the two solutions was adjusted to a molar ratio of Fe to NTA of 1:4 and a pH of 7.0 with sodium bicarbonate.

### Animal experiments

The Animal Care Committee of Okayama University Graduate School of Medicine, Dentistry and Pharmaceutical Sciences approved these experiments. The care and handling of the animals were in accordance with the National Institutes of Health Guidelines. Male Wistar rats (4 weeks old) were purchased from Japan SLC (Hamamatsu, Japan). They were housed in a temperature-controlled environment (25°C with alternating 12-h light/12-h dark cycles) and were allowed free access to distilled water and either a standard powdered-chow diet (MF) or a VE-deficient diet throughout the experiment. Ax-C-8 was dissolved in VE-stripped corn oil prior to use. VE-stripped corn oil was added to the basal diet [0.5% (v/w)] as a vehicle and fed to the Ax-C-8 groups.

Twenty-one rats (60–80 g) were used for the following pharmacological safety experiments (Table 1). The animals were fed by forced administration of Ax-C-8 with a gastric tube to monitor the effects on growth. In this experiment, the rats were divided into the following four groups: vehicle (*n* = 4), Ax-C-8 5 mg/animal/day (*n* = 4), Ax-C-8 25 mg/animal/day (*n* = 4) and Ax-C-8 49 mg/animal/day (*n* = 9). The rats were treated 5 days a week for four weeks. For the investigation of the antioxidant properties of Ax-C-8 in a regular diet, the rats were randomly divided into the following 3 groups: vehicle (*n* = 4), vehicle + Fe-NTA (*n* = 5–6), and Ax-C-8 (0.02%, w/w) + Fe-NTA (*n* = 5–6). These rats were fed a powdered diet with Ax-C-8 ad libitum for thirty days. Each animal received an i.p. injection of 9.0 mg Fe/kg of body weight using Fe-NTA solution. After the i.p. injection of Fe-NTA, the rats were euthanized 1, 4 and 24 h later, according to experiment design (Table 1). The sera were harvested, and the kidneys were transversely cut including the renal pelvis at a thickness of 5 mm and immediately fixed in 10% phosphate-buffered formalin. The rest of the renal tissues were cut into small pieces, snap frozen and kept at −80°C for further enzymatic examinations. In the experiments involving VE-deficient diets, twenty-eight rats were used (Table 1). The rats were divided into the following 5 groups: vehicle (*n* = 4), vehicle + Fe-NTA (*n* = 6), Ax-C-8 (0.01%) + Fe-NTA (*n* = 6), Ax-C-8 (0.02%) + Fe-NTA (*n* = 6), and Ax-C-8 (0.04%) + Fe-NTA (*n* = 6). All of the rats were fed a VE-deficient diet with or without Ax-C-8 for 2 weeks to induce VE deficiency. Fe-NTA was injected 3 times a week for four weeks. During the first week, the rats were treated with i.p. injections of 1.0 mg Fe/kg of body weight of Fe-NTA solution. From the second to fourth weeks, the rats received i.p. injections of 3.0 mg Fe/kg of body weight. The rats were euthanized at the end of the fourth week.

### Determination of the levels of 2-thiobarbituric acid-reactive substances

Lipid peroxidation was assessed based on the production of TBARS.^(15,24)^ In brief, 200 µl of postmitochondrial supernatants were added to a 2 ml mixture of trichloroacetic acid (5%, v/v) and TBA (0.34%, w/v). The samples were then heated to 95°C for 30 min. After centrifugation at 2,000 *g* for 10 min, the supernatants were measured using a spectrophotometer at 535 nm.

### Determination of calcium, creatinine and blood urea nitrogen levels

Calcium, creatinine and blood urea nitrogen (BUN) levels in sera were measured using an auto-analyzer (Hitachi 7600-110S).

### Determination of renal reduced glutathione and antioxidant enzyme activities

The enzymatic activities were assayed in the postmitochondrial supernatants of the renal homogenates.^(22,23)^ In brief, reduced glutathione was measured with DTNB as a chromogen. The enzymatic activities of glutathione peroxidase and glutathione reductase were measured using the method of NADPH oxidation in a coupled system. Catalase activity was measured using the method of H_2_O_2_ degradation. Glutathione S-transferase activity toward CDNB as a substrate was also measured.

### Immunohistochemical staining

 Immunohistochemical staining (IHC) was performed as previously described.^(22)^

### Statistical analysis

Statistical analyses were performed using one-way analysis of variance (ANOVA) and an unpaired *t* test. The differences were considered significant when *p*<0.05. These analyses were performed with GraphPad Prism5 Software (Graphpad, La Jolla, CA). For the animal studies, data are presented as SEM (*n* = 4–6) unless otherwise specified.

## Results

### The effects of Ax-C-8 on growth

We observed that a high oral dose (49 mg/animal/day) and a medium dose (25 mg/animal/day) affected the growth of some rats, but not all. In total, 4 out of 9 rats died within 5 days of the high dose experiment (Supplemental Fig. 1A*). The body sizes at death were 25–40 g, which is less than half the size of regular rats (Supplemental Fig. 1B*). In the medium dose experiment, 2 out of 4 rats died within 17 days (Supplemental Fig. 1A*). The autopsies involved macroscopic and microscopic examination found post-mortem changes. The rats that did not exhibit initial changes after receiving high and medium dosages of Ax-C-8 grew normally and were very active. In the experiment involving a low dose (5 mg/animal/day) and feeding with a mixed diet (0.02% w/w, *n* = 17) (Table 1), growth and activity were maintained in the rats.

### Determination of 2-thiobarbituric acid-reactive substances and reduced glutathione levels after Fe-NTA treatment

The TBARS levels were elevated 1 h after Fe-NTA treatment compared with the untreated animals (Vehicle 11.1 ± 0.5; Vehicle + Fe-NTA-1h, ^#^31.6 ± 1.35; Ax-C-8 + Fe-NTA-1h, ^#^30.2 ± 0.86; nmol/100 mg protein, *n* = 4–5, means ± SEM; ANOVA, *p*<0.0001, ^#^*p*<0.001 vs Vehicle). The renal levels of reduced GSH were remarkably depleted 1 h after administration of Fe-NTA with or without Ax-C-8 pretreatment. No antioxidative effects of Ax-C-8 were observed (Vehicle 22.1 ± 1.81; Vehicle + Fe-NTA-1h, ^#^11.1 ± 0.37; Ax-C-8 + Fe-NTA-1h, ^#^11.63 ± 0.39; Vehicle + Fe-NTA-4h, ^#^12.6 ± 0.78; Ax-C-8 + Fe-NTA-4h, ^#^13.29 ± 0.54; mmol/100 mg protein, *n* = 4–6, means ± SEM; ANOVA, *p*<0.0001, ^#^*p*<0.001 vs Vehicle).

### Ax-C-8 maintained renal function and antioxidative enzyme activities after Fe-NTA-induced oxidative injury

**Fig. 1:**
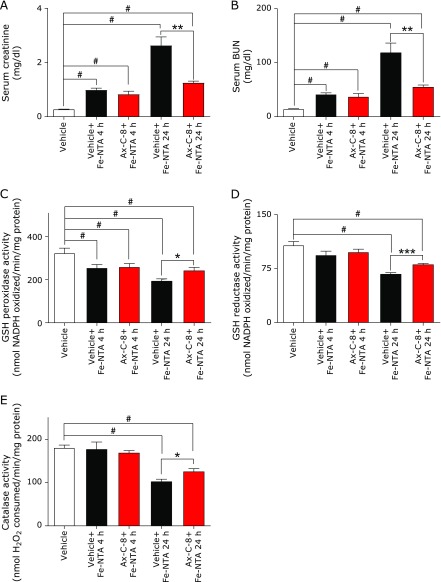
Treatment with astaxanthin C-8 (Ax-C-8) maintained renal function and antioxidant enzyme levels after ferric nitrilotriacetate (Fe-NTA)-induced oxidative injury. (A) Serum creatinine: Fe-NTA 4 h and 24 h, (B) serum BUN: Fe-NTA 4 h and 24 h. Protective effects of Ax-C-8 were observed, especially in the Fe-NTA 24 h group. (C) GSH peroxidase, (D) GSH reducase, (E) catalase. Attenuation of Fe-NTA-induced renal oxidative damage was observed in the Ax-C-8-treated group (ANOVA, *p*<0.0001, for a–e; ^#^*p*<0.05 vs vehicle; *p<0.05; **p<0.01 vs vehicle + Fe-NTA and ***p<0.001 vs vehicle + Fe-NTA).

An increase in the serum levels of creatinine and BUN was evident 4 and 24 h after Fe-NTA administration. Pretreatment with Ax-C-8 suppressed the elevations of these parameters at both time points (Fig. 1A and B). The activities of the antioxidative enzymes were decreased 4 h after Fe-NTA treatment, and pretreatment with the Ax-C-8 prevented this decrease, although the results were not statistically significant (*p*>0.05). Only the activity of GSH S-Transferase was significantly preserved after Ax-C-8 pretreatment (Vehicle 137.2 ± 5.54; Vehicle + Fe-NTA-4h, ^#^110.8 ± 2.61; Ax-C-8 + Fe-NTA-4h, *p<0.05, ^#^123.8 ± 6.09; nmol CDNB conjugate formed/min/mg protein, *n* = 4–6, means ± SEM; ANOVA, *p*<0.0001, ^#^*p*<0.001 vs vehicle; *p<0.05 vs Vehicle + Fe-NTA). Pretreatment with Ax-C-8 significantly maintained the levels of GSH reductase, GSH peroxidase and catalase 24 h after Fe-NTA-induced oxidative injury (Fig. 1C, D and E).

### Ax-C-8 suppressed renal tubular necrosis and the formation of HNE-modified proteins after Fe-NTA-induced oxidative injury in regular diet

**Fig. 2:**
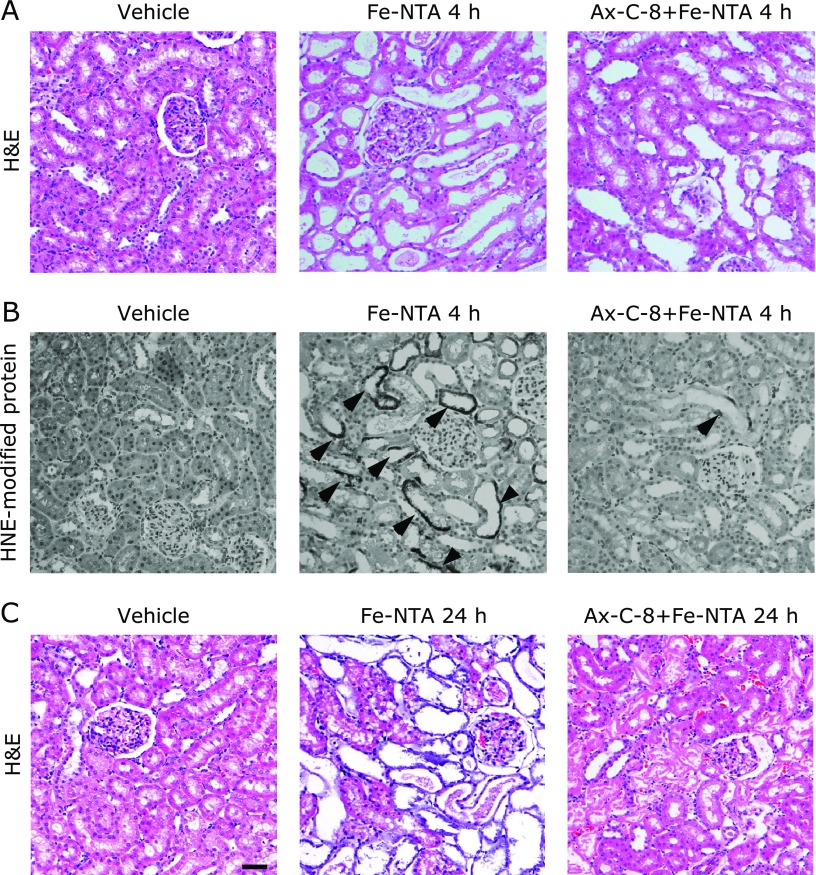
Astaxanthin C-8 (Ax-C-8) suppressed renal tubular necrosis and the formation of 4-hydroxy-2-nonenal (HNE)-modified proteins after Fe-NTA-induced oxidative injury in regular diet. Representative images are shown. (A) H&E staining of the vehicle, vehicle + Fe-NTA 4 h and Ax-C-8 + Fe-NTA 4 h groups. Many necrotic tubules were observed in the vehicle + Fe-NTA group, and Ax-C-8 suppressed the formation of necrotic tubules. (B) Immunohistochemical staining of HNE in the vehicle, vehicle + Fe-NTA 4 h and Ax-C-8 + Fe-NTA 4 h groups. HNE immunostaining revealed the accumulation of oxidatively modified proteins. Whereas no HNE-positive tubules were observed in the vehicle group, many tubules were positive (indicated by arrow head) in the Fe-NTA group. The levels of positive tubules were obviously decreased in the Ax-C-8 treated rats. (C) H&E staining of the vehicle, vehicle + Fe-NTA 24 h and Ax-C-8 + Fe-NTA 24 h groups. Fe-NTA destroyed the proximal tubules. However, pretreatment with Ax-C-8 protected against oxidative injury (bar, 50 µm).

The representative renal histology of the Fe-NTA-treated rats 4 h after Fe-NTA treatment is shown (Fig. 2A and B). Acute tubular necrosis was apparent in the proximal tubules, and the rats that were pretreated with Ax-C-8 were highly protected from tubular injury (Fig. 2A). In the case of the Fe-NTA-treated rats, HNE-modified proteins were observed in the degenerative proximal tubules, and the number of HNE-positive tubules was significantly decreased in the Ax-C-8-treated group. No positive tubules with apparent HNE-modified proteins were observed in the vehicle group (Fig. 2B). Massive destruction of the proximal tubules and cast depositions were observed in the vehicle + Fe-NTA group (Fig. 2C). These alterations were limited to localized areas by pretreatment with Ax-C-8 (Fig. 2C), and the oxidative stress was ameliorated 24 h after exposure to Fe-NTA in the Ax-C-8 group.

### Ax-C-8 accelerated the lethal injury mediated by Fe-NTA in VE-deficient conditions

**Fig. 3:**
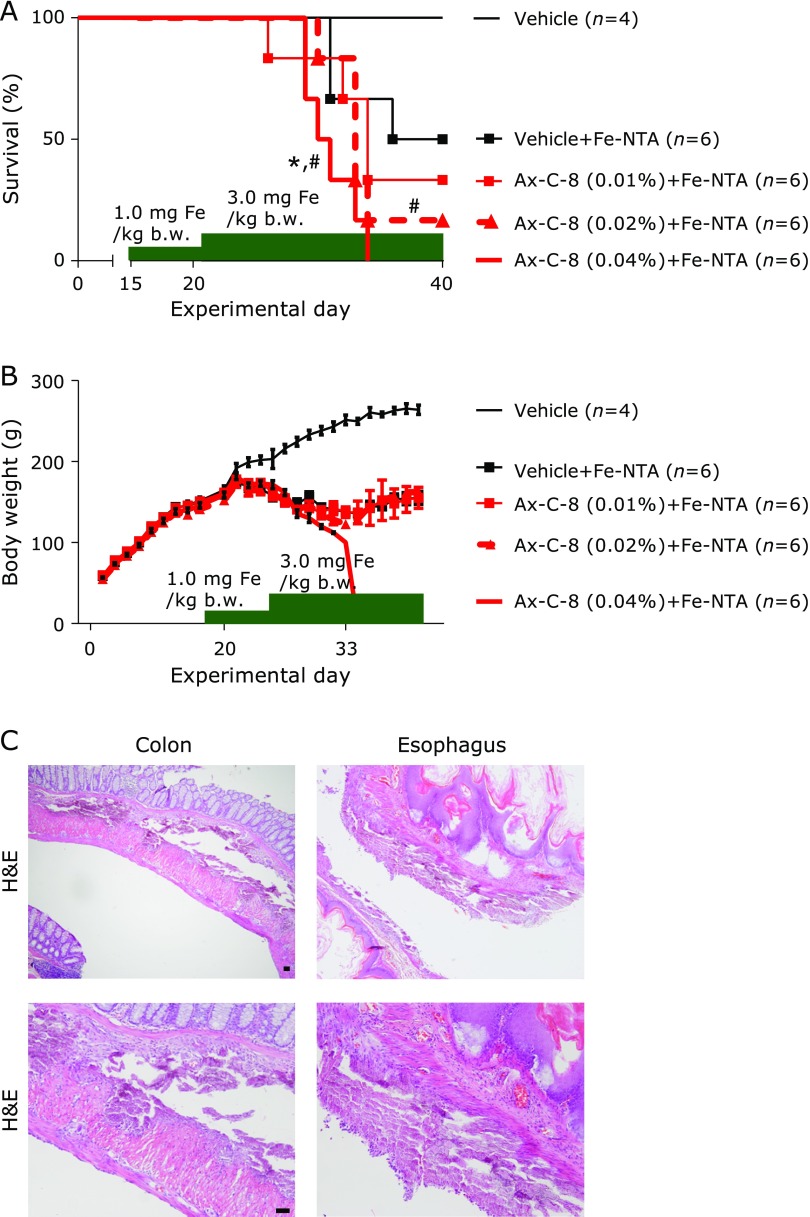
Astaxanthin C-8 (Ax-C-8) accelerated lethal injury mediated by Fe-NTA in Vitamin E (VE)-deficient conditions. (A) Survival curve of rats that received pretreatment with different concentrations of Ax-C-8 and a VE-deficient diet. Fe-NTA induced death in an Ax-C-8 dose-dependent manner (^#^*p*<0.05 vs vehicle; *p<0.05 vs vehicle + Fe-NTA for the log-rank test). (B) The body weights were decreased with repeated injections of Fe-NTA. Significant differences in body weights were not observed between the vehicle + Fe-NTA and Ax-C-8 + Fe-NTA groups. Fe-NTA induced massive diarrhea in the rats that were pretreated with Ax-C-8. (C) Microscopic examinations revealed intra-pleural and submucosal calcifications in the digestive tracts (bar, 50 µm).

During feeding with a VE-deficient diet containing Ax-C-8 to induce the depletion of VE, all of the rats were healthy. Fe-NTA injection (1.0 mg Fe/kg b.w.) did not result in any deaths during the first week, and Fe-NTA (3.0 mg Fe/kg b.w.) caused mortality that was dependent on the dosage of Ax-C-8 (Fig. 3A). During repeated injections of Fe-NTA, a loss of body weight was observed in the vehicle and Ax-C-8 groups (Fig. 3B), and diarrhea was observed in the Ax-C-8 group. Autopsies revealed intrapleural and submucosal calcification of the digestive tracts (Fig. 3C). These alterations were observed in the Ax-C-8-treated rats as follows: Ax-C-8 0.01%, 2/6 cases; 0.02%, 0/6 cases; and 0.04%, 1/6 cases. The serum concentrations of calcium, creatinine and BUN were not significantly different between the vehicle + Fe-NTA (*n* = 3) and Ax-C-8 (0.01%; *n* = 2, 0.02%; *n* = 1, 0.04%; *n* = 1) groups (Supplemental Table 1*).

## Discussion

In this study, we employed chemically modified Ax-C-8 to improve low bioavailability. The maximum tolerance of Ax-C-8 was analyzed, and a dosage of 25 mg/animal/day was found to induce death in rats. This dosage was equivalent to 300 mg/kg/day in humans and was nearly 1,000-fold higher than the ones used in CARET in Western countries and a clinical trial of astaxanthin in Japan.^(2,4)^ In the pharmacological safety test (Supplemental Fig. 1*), we attempted to detect Ax in the kidney using HPLC. However, Ax was not detected, even in the high concentration groups (Ax-C-8; 25 and 49 mg/animal/day) (data not shown).

Previous reports have suggested that a dose of 9.0 mg Fe/kg of body weight will induce an appropriate level of tissue injury to evaluate antioxidant compounds in rats.^(22,24)^ The antioxidant effects of Ax-C-8 (0.02%) against Fe-NTA were observed 4 h and 24 h after Fe-NTA treatment. Low concentrations of Ax-C-8 administered in a basal diet (0.005% and 0.01%) for 4 weeks were not able to protect against Fe-NTA-induced renal oxidative injury (data not shown). However, the antioxidant effects of Ax-C-8 (0.02%) were not striking 1 h after Fe-NTA treatment. Compared with previous reports, supplementation of VE-stripped corn oil has been shown to obviously worsen tissue damage. Supplementation with VE-stripped corn oil as a solvent for Ax-C-8 may accelerate tissue injury and increase the oxidant burden in the kidneys of rats. This may be why the early time points did not show any statistically significant protective effects after 1 h. Further studies of dietary oils using models of Fe-NTA-induced oxidative damage will be published elsewhere.

Ax-C-8 (0.02%) significantly suppressed Fe-NTA-induced renal tissue injury (Fig. 1 and 2), indicating that Ax-C-8 is an effective compound that prevents oxidative stress *in vivo*. Ax has also proven to be a promising chemopreventive compound against several types of carcinogens through the suppression of cellular proliferation,^(25–27)^ suggesting that an appropriate dosage of Ax-C-8 may be able to prevent Fe-NTA-induced renal carcinogenesis. However, antioxidants in human studies have not been effective, and this is presumed to be due to the nature of ROS, which can react with biomolecules immediately. Therefore, the use of antioxidants for chemoprevention is a subject that requires further trials of appropriate durations.^(28)^

In the VE-deficient experiment, we observed that supplementation with Ax-C-8 shortened survival in a dose-dependent manner. Although Ax and VE-derivative (tocotrienol) could demonstrate synergistic antioxidative effect,^(8)^ these data suggest that Ax is not appropriate for the replacement of VE. The combination of a VE-deficient diet and Fe-NTA may enhance iron-mediated oxidative injury. During treatment with Fe-NTA, some of the animals that were treated with Ax-C-8 developed diarrhea, as well as intra-pleural and submucosal calcifications (3/18 cases) (Fig. 3C). Ax has been shown to protect the skin, sera, salivary gland, liver, skeletal muscle, kidney and urinary bladder from oxidant,^(10–13,25,27)^ indicating that Ax is distributed throughout the whole body. Although only a small number of samples were available (3 samples from the vehicle + Fe-NTA group and 4 samples from the Ax-C-8 + Fe-NTA group), we collected sera and analyzed the serum concentrations of calcium. However, there were no differences between the vehicle and Ax-C-8 pretreatment groups with calcification and the Ax-C-8 pretreatment group without calcification (Supplemental Table 1*). Repeated injection of Fe-NTA caused osteopenia without significant changes in calcium, inorganic phosphorus, alkaline phosphate and parathyroid hormone levels in the rats fed a regular diet.^(29)^ A VE-deficient diet has been shown to impair bone calcification in rats,^(30)^ suggesting that VE is necessary for bone development. However, the combination of Fe-NTA and a VE-deficient diet without Ax-C-8 did not induce heterotopic calcification.^(14)^ The link between Fe-NTA and Ax-C-8 in bone metabolism must still be elucidated. In conclusion, we observed for the first time that Ax-C-8 protects against iron-mediated renal tubular injury in a rat model. Further study is warranted to determine the pathological conditions for the chemoprevention of oxidative stress.

## Figures and Tables

**Table 1:** Experimental design for rats in this study

Purpose	Treatment	Number of rats
Pharmacological safety	Vehicle	4
	Ax-C-8 (5 mg/day)	4
	Ax-C-8 (25 mg/day)	4
	Ax-C-8 (49 mg/day)	9

Antioxidant property against acute renal oxidative injury in basal diet (Ax-C-8; 0.02%, w/w)	Vehicle	4
	Vehicle + Fe-NTA 1 h	5
	Ax-C-8 + Fe-NTA 1 h	5
	Vehicle + Fe-NTA 4 h	5
	Ax-C-8 + Fe-NTA 4 h	6
	Vehicle + Fe-NTA 24 h	6
	Ax-C-8 + Fe-NTA 24 h	6

Antioxidant property against subacute renal oxidative injury in Vitamin E-deficient diet	Vehicle	4
	Vehicle + Fe-NTA	6
	Ax-C-8 (0.01%) + Fe-NTA	6
	Ax-C-8 (0.02%) + Fe-NTA	6
	Ax-C-8 (0.04%) + Fe-NTA	6

## Abbreviations

ANOVA: analysis of variance
Ax: astaxanthin
BUN: blood urea nitrogen
CDNB: 1-chloro-2,4-dinitrobenzene
DTNB: 5,5'-dithio-*bis*-2-nitrobenzoic acid
Fe-NTA: ferric nitrilotiriacetate
GSH: glutathione reduced form
HNE: 4-hydroxy-2-nonenal
HRP: horseradish peroxidase
NADPH: nicotinamide adenine dinucleotide phosphate reduced form
8-OHdG: 8-hydroxy-2'-deoxyguanosine
ROS: reactive oxygen species
RNS: reactive nitrogen species
TBARS: 2-thiobarbituric acid reactive substances
VE: vitamin E
