# Supplementary material for: Astaxanthin ameliorates ferric nitrilotriacetate-induced renal oxidative injury in rats
Source: J Clin Biochem Nutr. 2017 Jun 15;61(1):18–24. doi: 10.3164/jcbn.16-114 (PMC5525010; doi:10.3164/jcbn.16-114)
Supplement: Supplemental Fig. 1 [file jcbn16-114sf01.pdf]

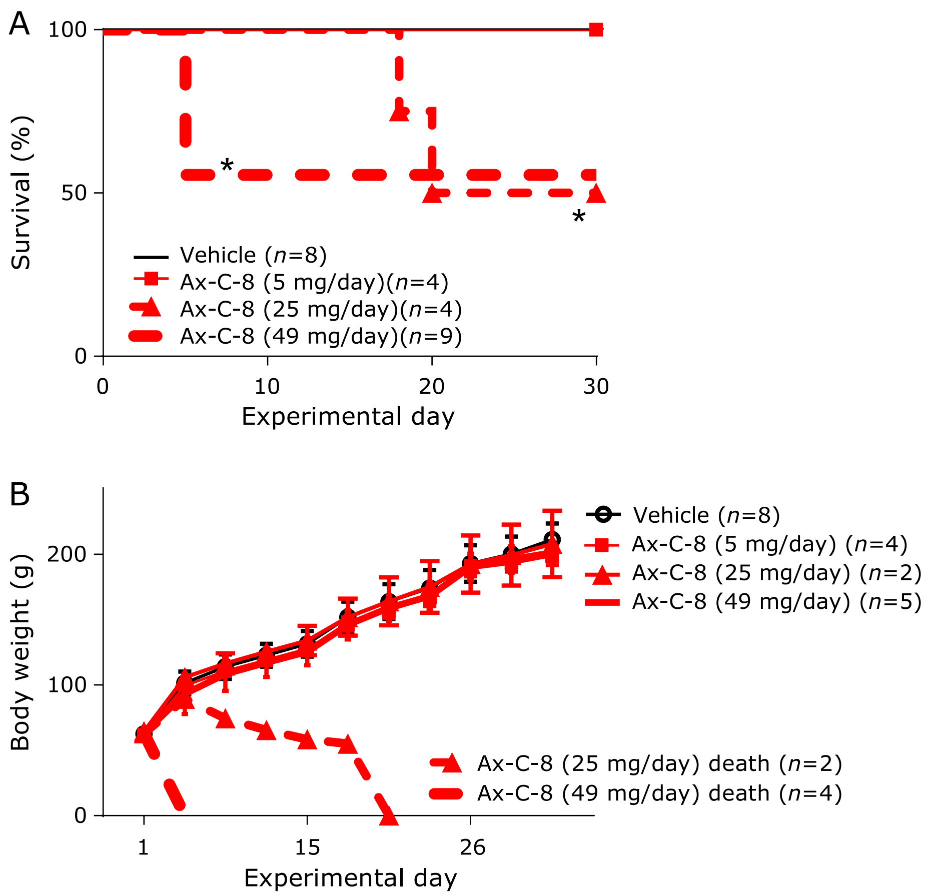

Supplemental Fig. 1. The effects of astaxanthin C-8 (Ax-C-8) on normal growth. (A) Forced feeding with Ax-C-8 at a high dose (49 mg/day) caused early death in 4 out of 9 rats by Day 4. A medium dose (25 mg/day) of Ax-C-8 led to growth retardation in half of the rats (2/4), which were found dead on Days 18 and 20. In the low dose group (5 mg/day), none of the rats exhibited retardation or abnormal behavior. (B) The body weights were maintained in the healthy rats, but some of the rats were debilitated with weight loss.
