# Supplementary material for: Astaxanthin ameliorates ferric nitrilotriacetate-induced renal oxidative injury in rats
Source: J Clin Biochem Nutr. 2017 Jun 15;61(1):18–24. doi: 10.3164/jcbn.16-114 (PMC5525010; doi:10.3164/jcbn.16-114)
Supplement: Supplemental Table 1 [file jcbn16-114st01.pdf]

Supplemental Table 1. Serum concentrations of calcium, creatinine and BUN in VE-deficient condition

Case No.	Treatment	Calcification	Calcium (mg/dl)	Creatinine (mg/dl)	BUN (mg/dl)
1	Vehicle + Fe-NTA	none	11.7	0.83	45.2
2	Vehicle + Fe-NTA	none	11.0	1.12	53.5
3	Vehicle + Fe-NTA	none	11.2	0.80	31.5
4	Ax-C-8 (0.01%) + Fe-NTA	present	11.2	0.75	22.7
5	Ax-C-8 (0.01%) + Fe-NTA	none	12.5	1.01	58.3
6	Ax-C-8 (0.02%) + Fe-NTA	none	13.3	0.66	29.1
7	Ax-C-8 (0.04%) + Fe-NTA	present	N/A	2.48	222.6
